# Rhizomelic Chondrodysplasia Punctata Type 1 Caused by a Novel Mutation in the PEX7 Gene

**DOI:** 10.4274/jcrpe.1835

**Published:** 2015-03-05

**Authors:** Abdullah Çim, Salih Coşkun, Orhan Görükmez, Hatice Yüksel, Ünal Uluca, Erminia Di Pietro, François Plourde, Nancy Elise Braverman

**Affiliations:** 1 Dicle University Faculty of Medicine, Department of Medical Genetics, Diyarbakır, Turkey; 2 Şevket Yılmaz Training and Research Hospital, Clinic of Medical Genetics, Bursa, Turkey; 3 Dicle University Faculty of Medicine, Department of Biochemistry, Diyarbakır, Turkey; 4 Dicle University Faculty of Medicine, Department of Pediatrics, Diyarbakır, Turkey; 5 McGill University and the Research Institute of the MUHC, Department of Pediatrics and Human Genetics, Quebec, Canada

**Keywords:** PEX7, novel, mutation, peroxisomal disorders, chondrodysplasia punctata, rhizomelic

## Abstract

Peroxisomes are involved in various metabolic reactions. Rhizomelic chondrodysplasia punctata (RCDP) type 1 is one of the peroxisomal biogenesis disorders caused by mutations in the PEX7 gene and is inherited in an autosomal recessive manner. We present a nine-year-old boy with skeletal abnormalities and dysmorphic facial appearance. The patient was born to parents who were first cousins. Very-long-chain fatty acids and pristanic acid levels were in the normal range, but an elevated phytanic acid level was detected by gas chromatography/mass spectrometry. The PEX7 gene was sequenced in the patient and his parents. A novel homozygous mutation, c.192delT (p.F64Lfs*10), was identified in the patient and was present in heterozygosity in both parents. In conclusion, the clinical presentation and peroxisome profile of the patient suggest that this novel mutation leads to RCDP type 1.

## INTRODUCTION

Rhizomelic chondrodysplasia punctata (RCDP) type 1 (OMIM, #215100) is an autosomal recessive disorder, characterized by disproportionally short stature due to the skeletal abnormalities including proximal shortening of the humerus and to a lesser degree the femur (rhizomelia); punctate calcifications in cartilage with epiphyseal and metaphyseal abnormalities (chondrodysplasia punctata) which can be seen on X-rays, joint deformities (congenital contractures); distinctive facial appearance including a prominent forehead, broad nasal bridge, hypertelorism, epicanthus, micrognathia, high arched palate and dysplastic external ears. Cataracts are found at birth or develop in early infancy in RCDP type 1. Intellectual disability is severe and most patients also develop seizures and spasticity (1).

Peroxisomes are membrane-enclosed ubiquitous subcellular organelles that contain many enzymes for various metabolic reactions. RCDP type 1 is one of the peroxisome biogenesis disorders and the defective gene responsible, PEX7, encodes the cytosolic receptors required for the transport of a few peroxisome matrix proteins (enzymes) into the peroxisomes (2). Peroxisome matrix proteins contain specific peroxisome targeting signals (PTS) including PTS1 and PTS2 on the primary peptide sequences. Most matrix proteins contain PTS1 and a few matrix proteins contain PTS2 (2). After the synthesis of these matrix proteins, their transport to the peroxisomes is mediated by PEX proteins, or peroxins, which are encoded by PEX genes (3). A cytosolic receptor, PEX5, binds the PTS1 signals and plays a role in the transfer and translocation of those peroxisomal matrix proteins into the peroxisomes. PEX7, also a cytosolic receptor, binds the PTS2 signals of peroxisomal matrix proteins, but also PEX5, for transfer to the peroxisomal membrane and translocation into peroxisome (4). PTS2-containing enzymes play a role in the beta-oxidation of fatty acids (3-ketothiolase), in the alpha-oxidation of fatty acids (phytanoyl-CoA hydroxylase) and in the biosynthesis of ether phospholipids including plasmalogens (alkyl-DHAP synthase, or AGPS) (2). The defective peroxin in RCDP type 1 is PEX7. Over 130 probands with RCDP1 have been reported and around 50% of the PEX7 mutations described are PEX7-L292*, a founder allele in Northern Europeans (5,6). Thus, it is important to describe PEX7 alleles in patients of non-European ancestry in order to record the full spectrum of PEX7 mutations. In this case report, we report the case of a patient from the Southeastern part of Turkey with RCDP in whom a novel mutation in the PEX7 gene was identified.

## CASE REPORT

A nine-year-old boy was referred to the Department of Medical Genetics for growth and developmental delay. The parents were first cousins and he was the first child of the family. He was born at term by Cesarean section due to breech position. Bilateral congenital cataracts (operated on at age two years), cryptorchidism on the right side, hypertonia and seizures were observed after birth. However, no further seizures occurred for about twelve months. The patient was reported to have severe developmental delay. He never learned to crawl or walk and did not say any words. He was fed by nursing bottle. His height and weight were within the normal range at birth, but below -2 standard deviations at all times after age 2 months.

**Figure 1:**
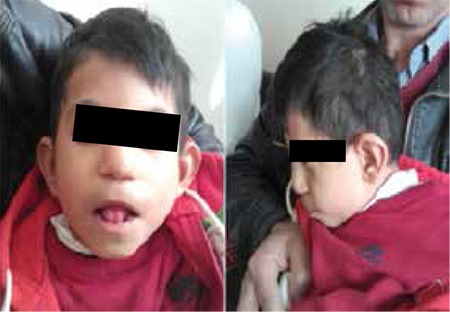
Picture of the patient. Dolichocephaly, flat occiput, maxillary hypoplasia, long- flattened-smooth philtrum, as well as wide nasal base and hypoplasia of the anterior nasal spine are features of the dysmorphic facial appearance

**Figure 2:**
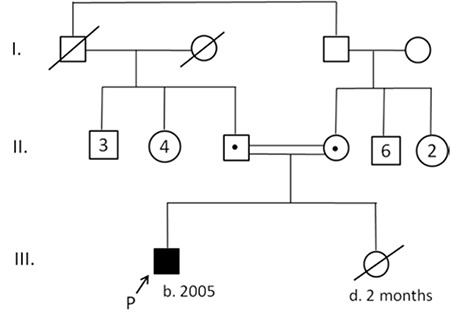
Pedigree of the family. The affected boy with RCDP is the first child of the family. The second baby died at age two months (b: date of birth, d: deceased)

Rhizomelic shortening of the arms, contractures at elbows and wrists, dolichocephaly, a flat occiput, maxillary hypoplasia, a long flat philtrum, a wide nasal base and hypoplasia of the anterior nasal spine were the dysmorphic features noted in the patient (Figure 1). His younger sibling had died following surgery for kidney disease at age 2 months, but this sibling did not have the clinical features of RCDP (see pedigree, Figure 2). Atrial septal defect (ASD) (3x3.5 mm), a left to right shunt, a small secundum ASD and peripheral pulmonary stenosis on the left side were detected by echocardiography at age 2 years. Cranial computerized tomography performed at age 3 years was reported as normal. Digital electroencephalography (EEG) was performed at age 5 years when he was awake and resting. No focal or paroxysmal abnormality was noted on the EEG. Liver, portal vein, hepatic vein, gallbladder, spleen, kidneys and adrenal glands were all reported as normal in an abdominal ultrasonography performed at age 6 years.

**Figure 3:**
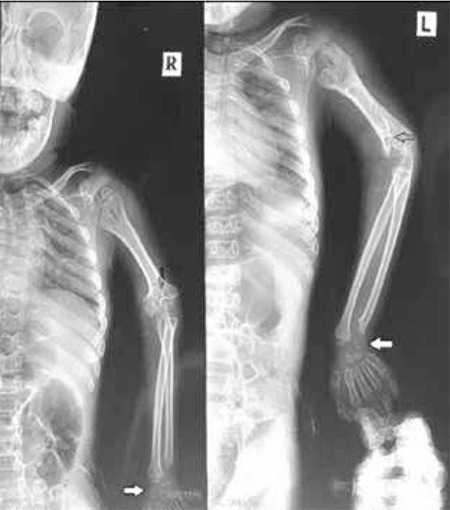
X-ray appearance of the upper extremities showing shortening of the humerus and calcifications at the elbow (black arrows). Ulnar hypoplasia and distal radio-ulnar dysplasia are present in both hands (white arrows). L: Left side, R: Right side of the patient

**Figure 4:**
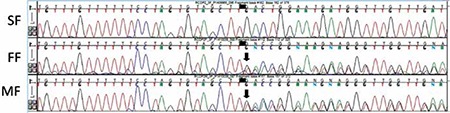
Chromatograms showing the mutation identified in the family. PEX7 gene was analyzed in the patient and his parents. The patient was homozygous for c.192delT (p.F64Lfs*10) in exon 3, and the parents were the carriers of this mutation. The top panel (SF) shows the patient’s sequencing result. The lower two panels (FF: father and MF: mother) show the heterozygous deletion in the parents (down black arrows). Homozygous deletion for c.192delT was confirmed by comparing the result with the RefSeq gene PEX7 (NM_000288.3)

At presentation, the patient was 9 years old. Rhizomelic shortening of the arms and calcifications at the elbow were observed. Ulnar hypoplasia and distal radio-ulnar dyplasia were detected on the X-rays (Figure 3). Gas chromatography/mass spectrometry (Shimadzu GCMS QP 2010 SE) performed at Dicle University revealed the blood levels of very-long-chain fatty acids (VLCFA) as C22: 68.7 µmol/L (N: 0-96.3 µmol/L), C24: 61.5 µmol/L (N: 0-91.4 µmol/L), C26: 0.8 µmol/L (N: 0-1.3 µmol/L) and pristanic acid 0.12 µmol/L (N: 0-2.98 µmol/L), values which were in the normal range (normal levels given in parenthesis). Phytanic acid level was 125.85 µmol/L (N: 0-9.88 µmol/L), a value which was very high. We elected to sequence the PEX7 gene in the patient and his parents. Ethylenediaminetetraacetic acid blood samples were sent to the Research Institute of the McGill University Health Centre in Montreal, Canada, in the context of a research protocol (7). Molecular genetic analysis was performed by Sanger sequencing of polymerase chain reaction amplicons of all 10 PEX7 exons and flanking intronic regions according to reported methods (5). The results indicated that the patient was homozygous for an unreported PEX7 variant, c.192delT (p.F64Lfs*10). This variant was found in the heterozygote state in both parents (Figure 4). It was not found in public exomes databases and is considered highly likely to be pathogenic and consistent with the diagnosis of RCDP type 1. The parents were informed about this disease and genetic counseling was provided. The family gave informed consent to use the X-rays, the patient’s photograph and test results for publication.

## DISCUSSION

The peroxisomal disorders are a clinically and genetically heterogeneous group of disorders in which there is impairment in peroxisome biogenesis or in the function of single peroxisomal enzymes. The peroxisome biogenesis disorders (PBDs) can be divided into two groups, the Zellweger syndrome spectrum disorders (ZSD) and RCDP type 1 (8). RCDP type 1 is clinically distinct from the ZSD and is characterized by rhizomelia, multiple punctuate epiphyseal calcification, cataracts, facial dysmorphism, microcephaly, small stature and psychomotor retardation (9).

The clinical diagnosis of a PBD can be suspected by biochemical laboratory assays. Elevated plasma VLCFA levels reflect deficient peroxisomal fatty acid metabolism. Elevated plasma levels of phytanic acid, pristanic acid, pipecolic acid and the bile acid precursors trihydroxycholestanoic acid and dihydroxycholestanoic acid and lowered concentrations of C16 and C18 plasmalogen levels in erythrocytes are also observed in PBDs and reflect other deficient peroxisome pathways. Following the biochemical assays, ideally, molecular testing and/or peroxisome enzymatic studies in primary skin fibroblasts are required to confirm the diagnosis (3).

Defects in any one of 13 PEX genes cause ZSD, but only defects in PEX7 cause RCDP type1. Nevertheless, the RCDP clinical phenotype can also be caused by single peroxisome enzyme defects in the pathway of plasmalogen synthesis. These enzymes are DHAP-AT (GNPAT) and alkyl-DHAP synthase (AGPS), causing RCDP type 2 and type 3, respectively. Recently, an atypical RCDP phenotype was described in 3 probands with FAR1 deficiency, the peroxisomal enzyme that provides the substrate, a fatty alcohol, for plasmalogen synthesis (10).

In all types of RCDP, VLCFAs and most other peroxisome biochemical markers are normal, while plasmalogen levels are typically reduced. Thus, it is suggested that disturbed plasmalogen synthesis is responsible for the clinical phenotype. In RCDP1, because the defect is in the PEX7 transporter, there is also a secondary biochemical defect in phytanic acid oxidation, leading to elevated phytanic acid levels in patients on diets that are not phytanic acid restricted. In the situation of a suspected RCDP patient, elevated phytanic acid levels target RCDP1 and eliminate the other single enzymes involved in the plasmalogen synthesis (5,10,11).

In this patient, phytanic acid level was elevated, but C22:0, C24:0, C26:0 and pristanic acid levels were in normal range. Considering the distinct clinical phenotype of RCDP compared to other peroxisomal disorders, we focused first on the diagnosis of RCDP type 1. We were unable to determine plasmalogen levels at our center but expect these to be reduced.

In conclusion, we have described a novel mutation, c.192delT (p.F64Lfs*10), in PEX7 gene. The clinical and biochemical presentation of the patient and the homozygous deletion, c.192delT, suggest that this mutation leads to RCDP type 1.
